# Supplementary material for: Characterization of prevalent genetic variants in the Estonian Biobank body-mass index GWAS
Source: Nat Commun. 2025 Oct 8;16:8956. doi: 10.1038/s41467-025-64006-9 (PMC12508233; doi:10.1038/s41467-025-64006-9)
Supplement: Supplementary file 1 — Supplementary Information [file 41467_2025_64006_MOESM1_ESM.pdf]

Supplementary figures for: “*Characterization of prevalent genetic variants in Estonian Biobank body-mass index GWAS*”

by Erik Abner, Kanwal Batool, Nele Taba, Tiit Nikopensius, Kristi Läll, Anastasiia Alekseienko, Anders Eriksson, Joel Rämö, Hele Haapaniemi, Hanna Maria Kariis, Liis Haljasmägi, Urmo Võsa, Taavi Tillmann, Uku Vainik, Kelli Lehto, Hanna M. Ollila, Kai Kisand, Estonian Biobank Research Team, Tõnu Esko

The purpose of this document is to provide additional information and figures that are not included in the main manuscript.

Contents

1. Supplementary Figure 1
Trait and covariate distributions for EstBB BMI GWAS.

2. Supplementary Figure 2
Manhattan plot of GWAS summary statistics on EstBB BMI GWAS.

3. Supplementary Figure 3
Q-Q plot and lambda inflation factor of EstBB BMI GWAS.

4. Supplementary Figure 4
Validation of the eight novel fine-mapped SNVs with FinnGen data.

5. Supplementary Figure 5
LocusZoom scatterplots of PIGW:His235Gln, PTPRT:p.Arg1384His and POMC:p.Glu206.*

6. Supplementary Figure 6
Variant effects in different population groups.

7. Supplementary Figure 7
Relationship between educational attainment and BMI.

8. Supplementary Figure 8
Sanger sequencing results of POMC:p.Glu206.*

9. Supplementary Figure 9
Principal component analysis plot of POMC:p.Glu206 variant carriers by nationality.*

10. Supplementary Figure 10
ADGRL3 locus in GenomeBrowser

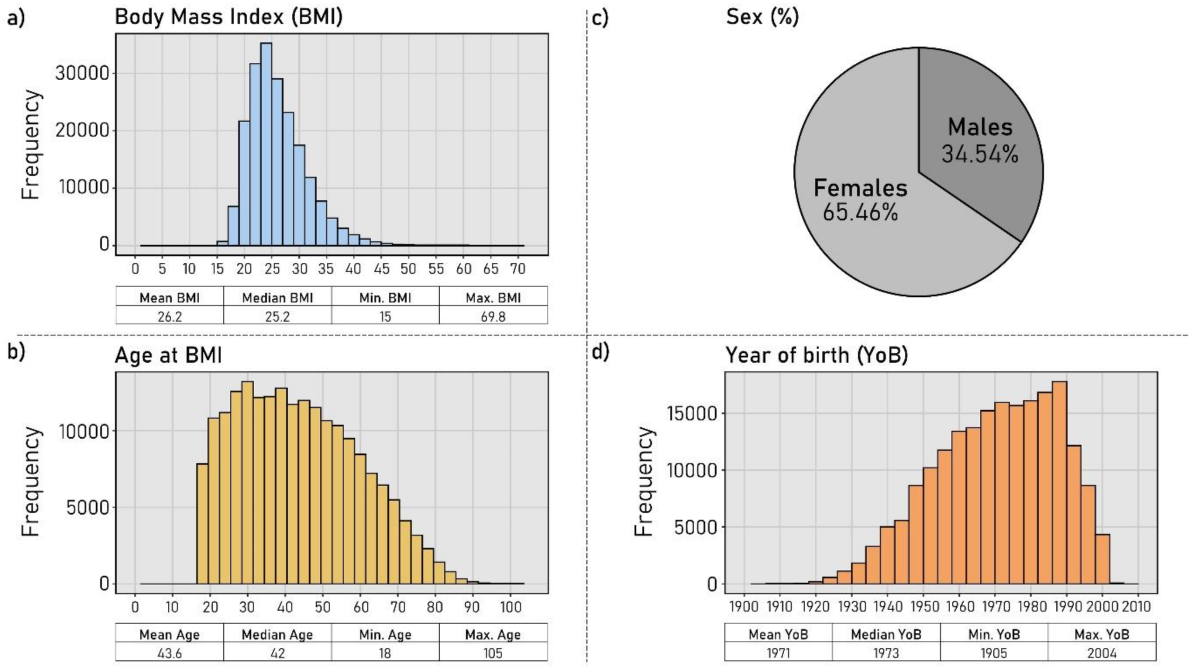

Supplementary Figure 1: Trait and covariate distributions for EstBB BMI GWAS.

a) *BMI distribution in the EstBB for earliest BMI per person (age ≥ 18). Note the slightly positively skewed distribution, which is why rank-based inverse-normal transformation was applied on the BMI variables.*

b) *Age distribution of the EstBB participants, at the age of BMI measurement.*

c) *Pie chart of male and female percentage distribution in the analyzed dataset.*

d) *Year of birth (YoB) was used as a covariate, as EstBB has been actively recruiting participants since 2003. Including YoB in the linear regression adjusts for temporal trends in BMI, ensuring data consistency across different collection periods.*

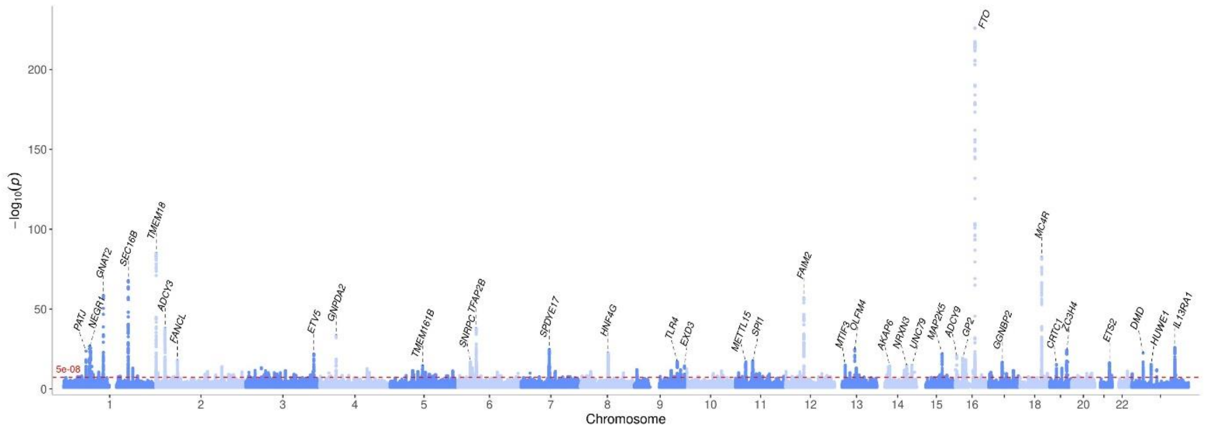

Supplementary Figure 2: Manhattan plot of GWAS summary statistics on EstBB BMI GWAS. *A total of 14,203,082 genetic variants (variants represented by round dots) were tested using two-sided linear regression, comparing 204,747 Estonian Biobank participants by BMI as a continuous trait and employing rank-based inverse normal transformation. The lead hits are in or near genes FTO (rs1421085; P = 1.74 × 10^{-205} ; +0.466kg/m^2; 95% CI = 0.436 – 0.496), MC4R (rs523288; P = 1.97 × 10^{-74}; +0.357kg/m^2; 95% CI = 0.319 – 0.395) and TMEM18 (rs35296565; P = 1.14 × 10^{-69}; +0.358kg/m^2; 95% CI = 0.318 – 0.397). The x axis represents the genome in physical order, and the y axis represents P values (–log10(P value)) of association. The red horizontal line represents the P value threshold of 5 × 10^{-8}. Loci with lead hits with P value below 1 × 10^{-15} have been highlighted.*

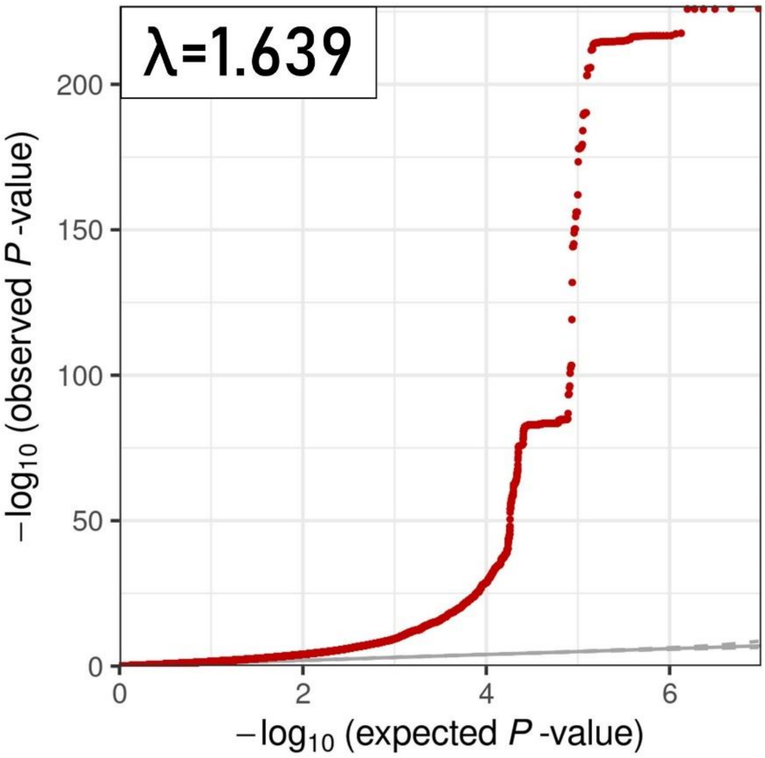

Supplementary Figure 3: Q-Q plot and lambda inflation factor of EstBB BMI GWAS. *The Q-Q plot shows the distribution of association based on observed (x axis) and expected (y axis) -log10 transformed P values under the null hypothesis of no association. The gray line shows the distribution of association for null hypothesis, while the red dots show actual distribution of association. Despite accounting for relatedness, the genomic inflation factor indicated considerable inflation (λs = 1.639). To understand whether this inflation was due to true polygenic effects or potential biases such as residual population substructure, we employed linkage disequilibrium score regression (LDSC). The analysis yielded an LDSC intercept of 1.1535 and a mean χ^2 of 2.1129. These values were used to partition the observed inflation into polygenic effects and biases. Specifically, the proportion of inflation attributable to biases was calculated using the LDSC ratio, defined as (intercept - 1) / (mean χ^2 - 1). In our study, the LDSC ratio was (1.1535 - 1) / (2.1129 - 1) ≈ 0.1379, indicating that biases accounted for approximately 14% of the inflation. Conversely, the polygenic effects contributed the remaining 86%, confirming that the majority of the inflation was due to true polygenic signals, leading us to conclude that additional correction was unnecessary.*

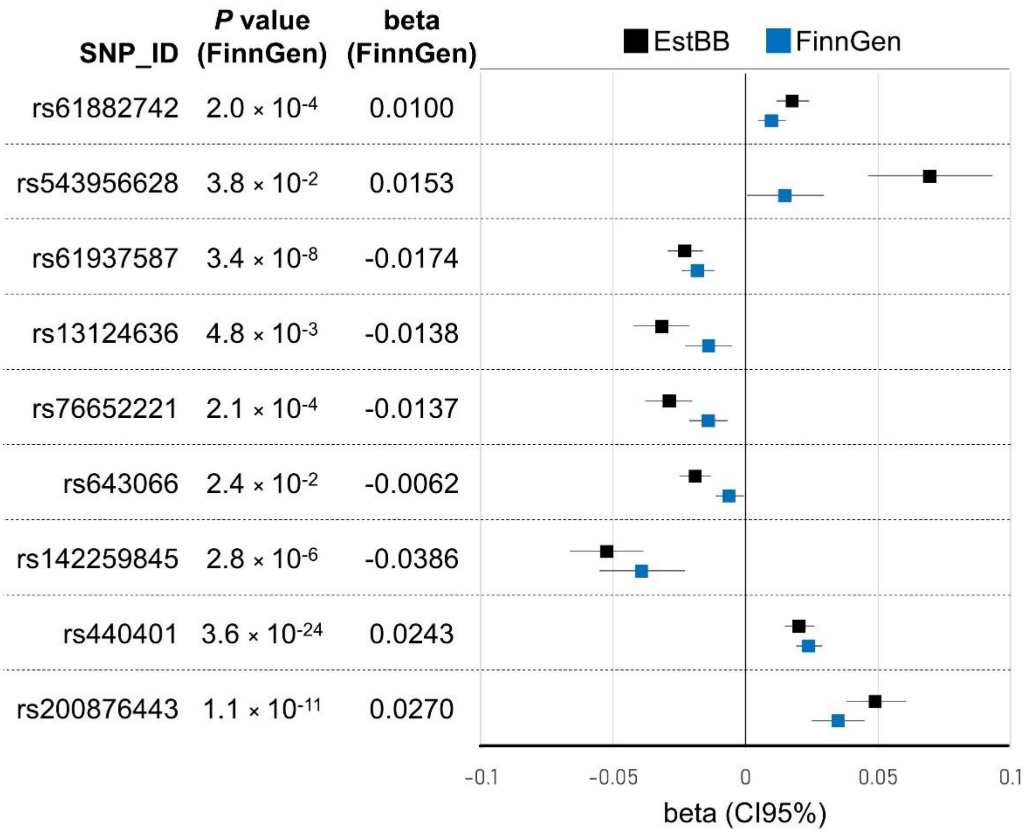

Supplementary Figure 4: Validation of the eight fine-mapped SNVs with FinnGen data. *Forest plot illustrating the effect sizes (β) of eight SNVs from FinnGen r10 freeze 'Body-mass index, inverse-rank normalized' GWAS (blue), based on a two-sided linear regression. The Estonian Biobank (EstBB) BMI GWAS is included as a comparison (black). Betas and P values are from rank-based inverse normal transformation (RINT) GWASs; error bars represent ± 95% confidence intervals. P values are exact and unadjusted. All data represent biological replicates; no technical replicates were used. The EstBB data originate from the same GWAS as presented in Figure 1. FinnGen GWAS included n = 290,820 participants and is publicly available at https://r10.finngen.fi/pheno/BMI_IRN.*

a)

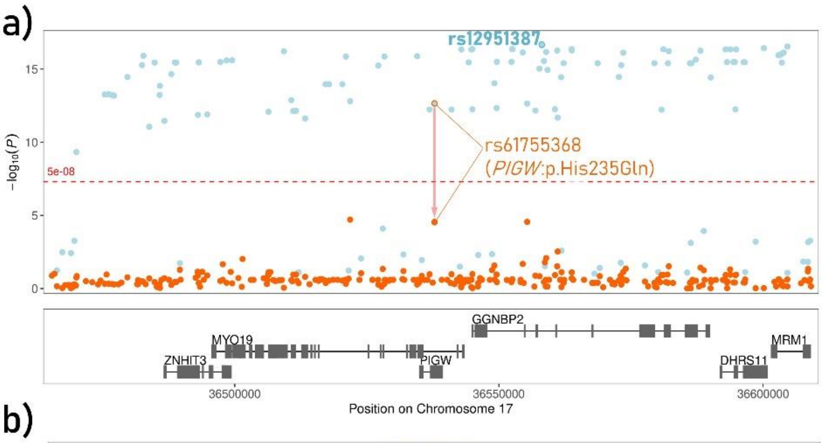

PIGW:p.His235Gln	beta	SE	P value
Pre-Conditional	-0.242	0.0385	3.26×10^{-10}
Conditional	-0.166	0.0397	2.78×10^{-5}

b)

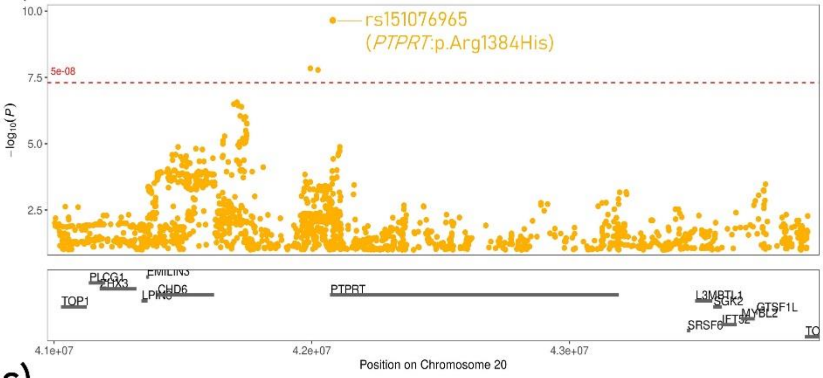

PTPRT:p.Arg1384His	beta	SE	P value
rs151076965	-0.4385	0.0693	2.51×10^{-10}

c)

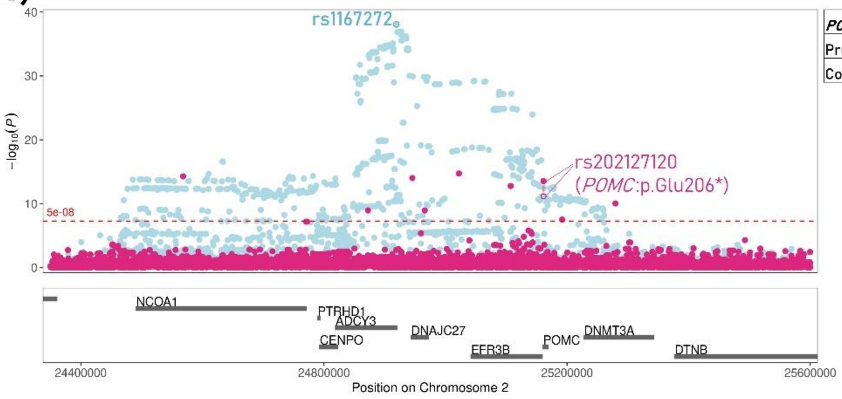

POMC:p.Glu206*	beta	SE	P value
Pre-Conditional	0.8092	0.1144	1.48×10^{-12}
Conditional	0.8689	0.1143	2.87×10^{-14}

Supplementary Figure 5: LocusZoom scatterplots of PIGW:His235Gln, PTPRT:p.Arg1384His and POMC:p.Glu206*.

a) *Scatterplot displaying the locus around PIGW:His235Gln from the BMI GWAS. Lead hit of the locus has been highlighted in blue. Orange dots display P values following the conditional analysis for the locus lead hit.*

b) *Scatterplot displaying the locus around PTPRT:p.Arg1384His from the BMI GWAS.*

c) *Scatterplot displaying the locus around POMC:p.Glu206* from the BMI GWAS. Lead hit of the locus has been highlighted in blue. Pink dots display P values following the conditional analysis for the locus lead hit.*

The red vertical arrows in panels a) and c) indicate the shift in P value following the conditional analyses.

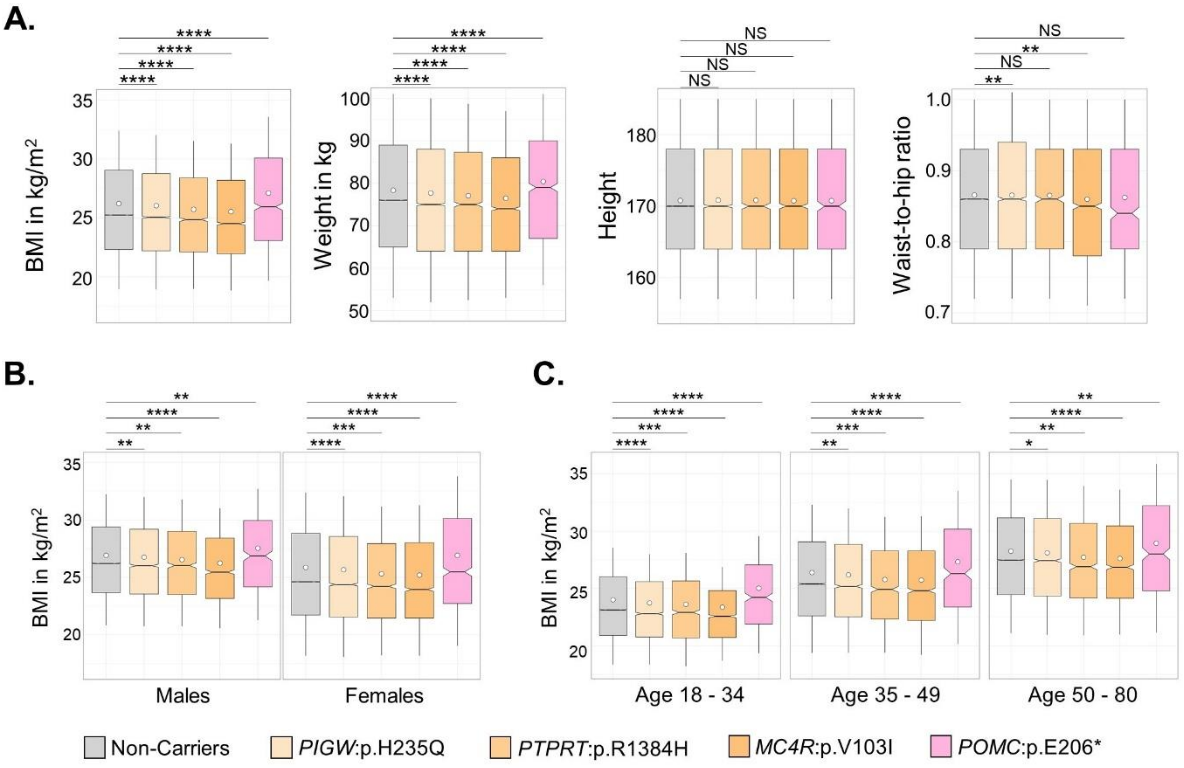

Supplementary Figure 6: Variant effects in different population groups.

A-C) *Box plots depicting the distribution of anthropometric traits (body mass index, height, weight, waist-to-hip ratio) across carrier groups of four coding SNVs and non-carriers. Each box represents the interquartile range (IQR), the horizontal line indicates the median, and the white dot shows the mean. Notches represent approximate 95% confidence intervals of the median. Whiskers extend to 0.5 × IQR, and outliers are omitted for clarity. Asterisks above boxes indicate statistical significance from two-sided linear regression comparing heterozygous carriers to non-carriers, performed using REGENIE (*P < 0.05; **P < 0.01; ***P < 0.001; ****P < 0.0001; NS = not significant). Exact P values and sample sizes per group are provided in the accompanying Source Data. P values are unadjusted. BMI – Body Mass Index; WHR – Waist-Hip Ratio.*

Colors: non-carriers (gray), POMC:p.Glu206 (pink), PIGW:His235Gln (light yellow), MC4R:p.Val103Ile (dark yellow), PTPRT:p.Arg1384His (yellow).*

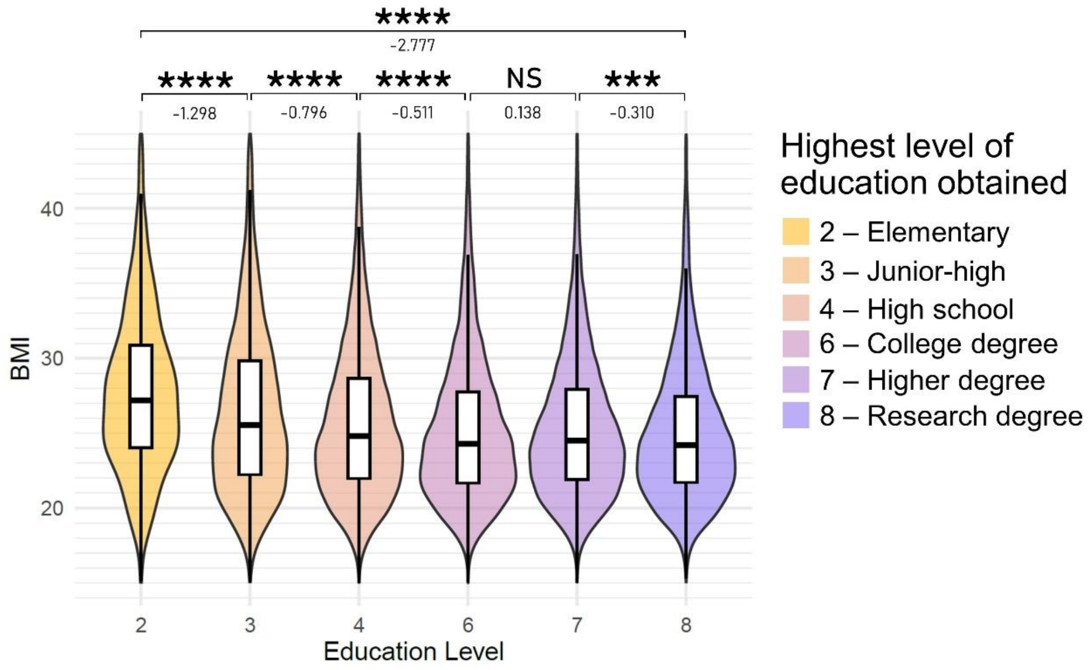

Supplementary Figure 7: Relationship between educational attainment and BMI.

*Violin plots showing the distribution of BMI across six educational attainment groups, from elementary education to research degree. Color gradient spans from yellow (lowest education) to purple (highest education). Each violin represents the full BMI distribution per group, with a central line marking the median and box indicating the interquartile range (IQR). Pairwise group comparisons were assessed using one-way ANOVA followed by Tukey's Honest Significant Difference (HSD) test. Asterisks above violins indicate significant group differences (*P < 0.05; **P < 0.01; ***P < 0.001; ****P < 0.0001). Exact P values and sample sizes per group are provided in the accompanying Source Data.*

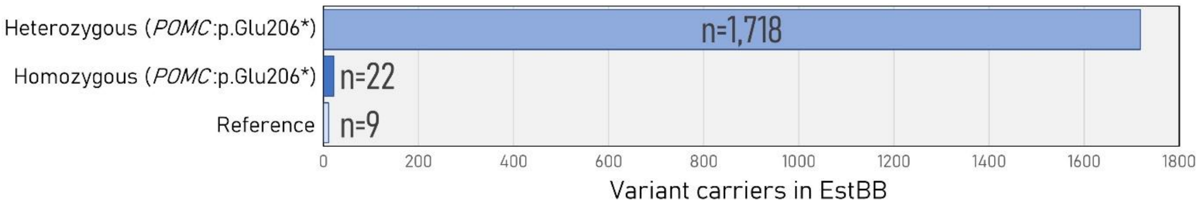

Supplementary Figure 8: Sanger sequencing results of POMC:p.Glu206*.

Bar plot displaying sequencing results of predicted POMC:p.Glu206 variant carriers in Estonian Biobank. Variant carriers were predicted using a local reference panel for imputation. The genotype distribution does not significantly deviate from Hardy-Weinberg equilibrium ($\chi 2=1.622$; $P = 0.203$), suggesting that there is no strong selection pressure against the variant, implying it is not deleterious in the population.*

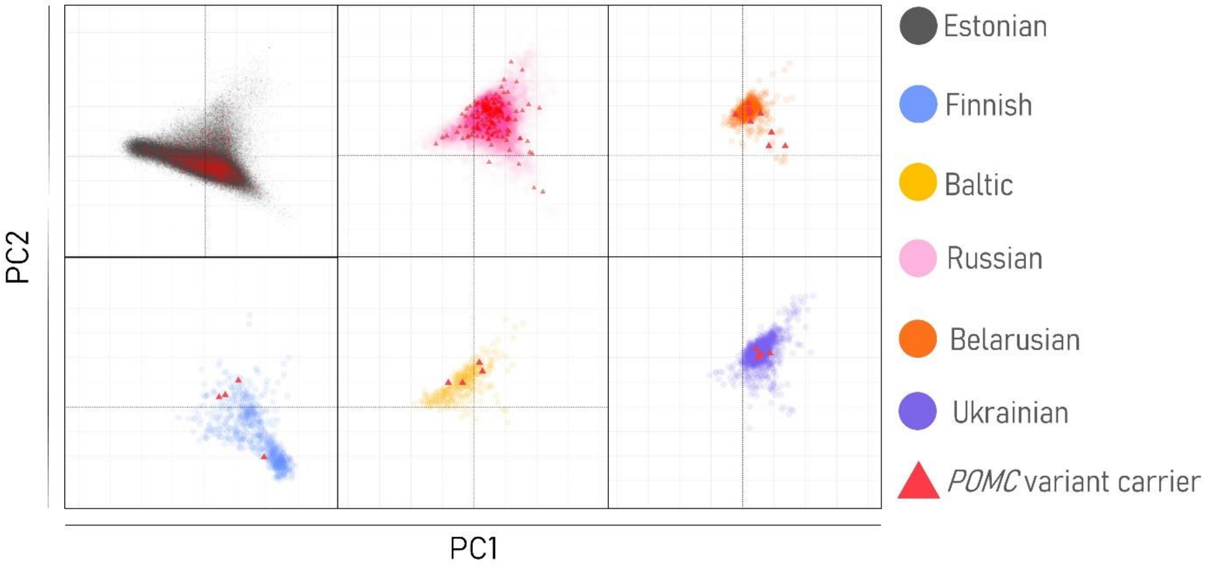

Supplementary Figure 9: Principal component analysis plot of POMC:p.Glu206* variant carriers by self-reported nationality.

Principal component analysis (PCA) plot showing genetic clustering of participants by self-reported nationality, with POMC:p.Glu206 variant carriers highlighted as red triangles. Colors indicate nationalities: Estonian (black), Russian (pink), Finnish (blue), Belarusian (orange), Ukrainian (purple), Latvian and Lithuanian (grouped as "Baltic" in yellow). Axes represent the first two genetic principal components (PC1 and PC2). Exact sample sizes per group are provided in the accompanying Source Data.*

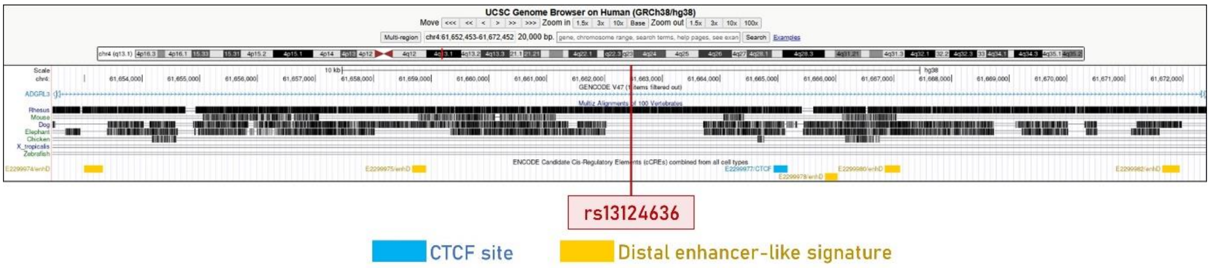

Supplementary Figure 10: ADGRL3 locus in GenomeBrowser

The graph depicts the locus around the lead hit rs13124636 (red) in chromosome 4q13.1, within ADGRL3 gene intron 5, highlighting the genomic features ±10 kilobases from the variant. This region contains 5 distal enhancer-like signatures (yellow) and a CTCF site (blue).

Image obtained on 16.12.2024 from: https://genome-euro.ucsc.edu/cgi-bin/hgTracks?db=hg38
