# Supplementary material for: Characterization of prevalent genetic variants in the Estonian Biobank body-mass index GWAS
Source: Nat Commun. 2025 Oct 8;16:8956. doi: 10.1038/s41467-025-64006-9 (PMC12508233; doi:10.1038/s41467-025-64006-9)
Supplement: Supplementary file 2 — Description of Additional Supplementary Files [file 41467_2025_64006_MOESM2_ESM.pdf]

Description of Additional Supplementary Files

Supplementary Data 1: Genetic correlation between EstBB BMI GWAS with previously published data using LDSC. LDSC regression was performed using the CTG-VL platform to estimate genome-wide genetic correlations between EstBB BMI GWAS and an agnostic panel of public GWAS summary statistics. The table lists the top 100 correlations ranked by P value. Analyses were two-sided, and P values are reported as exact and unadjusted. Datasets with known sample overlap were excluded. Rg – genetic correlation between traits.

Supplementary Data 2: List of all fine-mapped hits in EstBB BMI GWAS. Each listed SNV represents the variant with the highest posterior inclusion probability (PIP) within its 1 Mb genomic window. Fine-mapping was performed using the finemap.abf function from the coloc v5.2.3 R package, as implemented in our custom pipeline. Effect sizes (β), standard errors (SE), and P values are derived from two-sided linear regression models. PIP indicates the probability of the variant being causal. P values are exact and unadjusted.

Supplementary Data 3: List of the newly identified four SNVs with functional effects on protein structure. Effect sizes (β), standard errors (SE), and P values are derived from two-sided linear regression models using the non-RINT BMI phenotype to retain interpretability. INFO score indicates imputation quality. MAF – minor allele frequency. P values are exact and unadjusted.

Supplementary Data 4: PheWAS results for PTPRT:p.Arg1384His. This analysis was performed using a binomial logistic regression (glm) adjusting for age, age^2, sex, year of birth, and the first ten genetic principal components. Data are sorted by P value. Analyses were two-sided; effect sizes are presented as odds ratios (OR) with standard errors (SE) and exact, unadjusted P values. PheCode – phenotype code.

Supplementary Data 5: Variant effects based on different data sources and diagnostic exclusions.
“BMI_All” trait contains the original dataset (N=204,747).
“BMI_Cleaned” group contains the participant group, where no confounding diagnoses were observed (N= 131,991).
“BMI_HealthRecords” group contains BMI data only obtained from electronic health records (N=144,268).
“BMI_SelfReported” group contains BMI data only obtained from self-reported questionnaires (N= 201,168).
The list of exclusion factors for “BMI_Cleaned” was obtained from Orthofer, Michael, et al. "Identification of ALK in Thinness." Cell 181.6 (2020): 1246-1262.
Effect sizes were obtained by two-directional linear regression using the REGENIE.

Supplementary Data 6: PheWAS results for POMC:p.Glu206*. This analysis was performed using a binomial logistic regression (glm) adjusting for age, age^2, sex, year of birth, and the first ten genetic principal components. Data are sorted by P value. Analyses were two-sided; effect sizes are presented as odds ratios (OR) with standard errors (SE) and exact, unadjusted P values. PheCode – phenotype code.
