# Supplementary material for: Characterization of prevalent genetic variants in the Estonian Biobank body-mass index GWAS
Source: Nat Commun. 2025 Oct 8;16:8956. doi: 10.1038/s41467-025-64006-9 (PMC12508233; doi:10.1038/s41467-025-64006-9)
Supplement: Supplementary file 4 — Reporting Summary [file 41467_2025_64006_MOESM4_ESM.pdf]

nature portfolio

Corresponding author(s): Erik Abner
Kanwal Batool

Last updated by author(s): Aug 6, 2025

Reporting Summary

Nature Portfolio wishes to improve the reproducibility of the work that we publish. This form provides structure for consistency and transparency in reporting. For further information on Nature Portfolio policies, see our Editorial Policies and the Editorial Policy Checklist.

Statistics

For all statistical analyses, confirm that the following items are present in the figure legend, table legend, main text, or Methods section.

n/a	Confirmed	
☐	☒	The exact sample size (n) for each experimental group/condition, given as a discrete number and unit of measurement
☒	☐	A statement on whether measurements were taken from distinct samples or whether the same sample was measured repeatedly
☐	☒	The statistical test(s) used AND whether they are one- or two-sided<br>*Only common tests should be described solely by name; describe more complex techniques in the Methods section.*
☐	☒	A description of all covariates tested
☐	☒	A description of any assumptions or corrections, such as tests of normality and adjustment for multiple comparisons
☐	☒	A full description of the statistical parameters including central tendency (e.g. means) or other basic estimates (e.g. regression coefficient) AND variation (e.g. standard deviation) or associated estimates of uncertainty (e.g. confidence intervals)
☐	☒	For null hypothesis testing, the test statistic (e.g. F, t, r) with confidence intervals, effect sizes, degrees of freedom and P value noted<br>*Give P values as exact values whenever suitable.*
☒	☐	For Bayesian analysis, information on the choice of priors and Markov chain Monte Carlo settings
☒	☐	For hierarchical and complex designs, identification of the appropriate level for tests and full reporting of outcomes
☐	☒	Estimates of effect sizes (e.g. Cohen's d, Pearson's r), indicating how they were calculated

Our web collection on statistics for biologists contains articles on many of the points above.

Software and code

Policy information about availability of computer code

Data collection	No software was used collect data.
Data analysis	All software programs used for the analyses described in this paper are freely available online:<br>REGENIE v3.2 (https://github.com/rgcgithub/regenie); UCSC Genome Browser for SNV analysis (http://genome.ucsc.edu/); Statistical analysis were carried out using R version 4.2.1; FUMA v1.4.0 (https://fuma.ctglab.nl/); LDSC v1.0.1 (https://github.com/bulik/ldsc); PLINK2 (www.cog-genomics.org/plink/2.0/); protein dynamics were evaluated with DynaMut2 (https://biosig.lab.uq.edu.au/dynamut2/); visualization of the protein structures and docking results using ChimeraX v1.8 (https://www.rbvi.ucsf.edu/chimerax/); Fine-mapping (https://github.com/urmovosa/FinemapAbf/tree/main); PGS Catalog Calculator v2.0.0-beta.3 (https://github.com/PGScatalog/pgsc_calc); For FinnGen, code to perform GWAS analyses is available at the FinnGen GitHub (https://github.com/FINNGEN/). Individual plots were created using R v3.6.3, v4.2.2 and v4.3.2, including the R packages ggplot2, RColorBrewer, geojsonio, sf and topr. Map in Figure 4a was generated using publicly available GeoJSON data from the Natural Earth dataset. The final figures were edited with Microsoft Powerpoint.

For manuscripts utilizing custom algorithms or software that are central to the research but not yet described in published literature, software must be made available to editors and reviewers. We strongly encourage code deposition in a community repository (e.g. GitHub). See the Nature Portfolio guidelines for submitting code & software for further information.

Data

Policy information about availability of data

All manuscripts must include a data availability statement. This statement should provide the following information, where applicable:

- Accession codes, unique identifiers, or web links for publicly available datasets
- A description of any restrictions on data availability
- For clinical datasets or third party data, please ensure that the statement adheres to our policy

GWAS summary statistics are available on GWAS Catalog (https://www.ebi.ac.uk/gwas/home) under accession numbers GCST90624699; GCST90624700; GCST90624701;GCST90624702; GCST90624703; GCST90624704; GCST90624705. The procedure to access EstBB individual-level data has been described at https://genomics.ut.ee/en/content/estonian-biobank#dataaccess. For validation with FinnGen data freeze 10 was used (https://r10.finngen.fi/).
Source Data file contains all necessary data and additional supplementary data is provided in Supplementary Data file needed for replication of this study.

Research involving human participants, their data, or biological material

Policy information about studies with human participants or human data. See also policy information about sex, gender (identity/presentation), and sexual orientation and race, ethnicity and racism.

Reporting on sex and gender	Sex is defined based on genotype-inferred biological sex. Individuals with genotype and phenotype discrepancies were excluded.
Reporting on race, ethnicity, or other socially relevant groupings	All study participants are of European ancestry.
Population characteristics	Participants are of European ancestry, restricted to age 18 and above. All participant information is obtained from the national health insurance fund and electronic health records.
Recruitment	The Estonian Biobank is a volunteer-based biobank with 212,955 participants in the current data freeze [PMID:40188112]. All biobank participants have signed a broad informed consent form and health information is obtained via regular linking with the national Health Insurance Fund and other relevant databases, with majority of the electronic health records having been collected since 2004 [PMID:40188112].
Ethics oversight	Individual level data analysis in EstBB was carried out under ethical approval 1.1-12/624 from the Estonian Committee on Bioethics and Human Research (Estonian Ministry of Social Affairs), using data according to release application 3-10/GI/16856 from the Estonian Biobank.

Note that full information on the approval of the study protocol must also be provided in the manuscript.

Field-specific reporting

Please select the one below that is the best fit for your research. If you are not sure, read the appropriate sections before making your selection.

☒ Life sciences ☐ Behavioural & social sciences ☐ Ecological, evolutionary & environmental sciences

For a reference copy of the document with all sections, see nature.com/documents/nr-reporting-summary-flat.pdf

Life sciences study design

All studies must disclose on these points even when the disclosure is negative.

Sample size	Estonian biobank BMI dataset consisting of n=204,747 participants after applying necessary exclusion criteria were used for this study.
Data exclusions	Analyses were restricted to individuals with European ancestry. Participants below the age of 18, participants with BMI values below 15 kg/m2 and above 70 kg/m2, participants with sex definition discrepancies and pregnant individuals were excluded from the study.
Replication	The replication analysis of GWAS analysis involved multiple approaches: we performed systemic search of associated loci for BMI in various databases including GWAS Catalogue, and FinnGen for validity check-up and the directionality of the effect. We also performed a comprehensive literature review to find the supporting evidence from animal model studies (mentioned extensively in the manuscript).
Randomization	No randomization was performed. To control for potential stratification age/year of birth and first 10 genetic principal components were used as covariates in GWAS.
Blinding	All samples and personal information was pseudonymous.

Reporting for specific materials, systems and methods

We require information from authors about some types of materials, experimental systems and methods used in many studies. Here, indicate whether each material, system or method listed is relevant to your study. If you are not sure if a list item applies to your research, read the appropriate section before selecting a response.

Materials & experimental systems

n/a	Involved in the study
☒	☐ Antibodies
☒	☐ Eukaryotic cell lines
☒	☐ Palaeontology and archaeology
☒	☐ Animals and other organisms
☒	☐ Clinical data
☒	☐ Dual use research of concern
☒	☐ Plants

Methods

n/a	Involved in the study
☒	☐ ChIP-seq
☒	☐ Flow cytometry
☒	☐ MRI-based neuroimaging

Plants

Seed stocks	*Report on the source of all seed stocks or other plant material used. If applicable, state the seed stock centre and catalogue number. If plant specimens were collected from the field, describe the collection location, date and sampling procedures.*
Novel plant genotypes	*Describe the methods by which all novel plant genotypes were produced. This includes those generated by transgenic approaches, gene editing, chemical/radiation-based mutagenesis and hybridization. For transgenic lines, describe the transformation method, the number of independent lines analyzed and the generation upon which experiments were performed. For gene-edited lines, describe the editor used, the endogenous sequence targeted for editing, the targeting guide RNA sequence (if applicable) and how the editor was applied.*
Authentication	*Describe any authentication procedures for each seed stock used or novel genotype generated. Describe any experiments used to assess the effect of a mutation and, where applicable, how potential secondary effects (e.g. second site T-DNA insertions, mosiacism, off-target gene editing) were examined.*
